# Attitudes towards the use of perioperative steroids in resectional colorectal cancer surgery in the UK: A qualitative study

**DOI:** 10.1016/j.amsu.2019.10.007

**Published:** 2019-10-11

**Authors:** Allan M. Golder, Stephen T. McSorley, Rachel J. Kearns, Donald C. McMillan, Paul G. Horgan, Campbell S. Roxburgh

**Affiliations:** Academic Unit of Surgery, Glasgow Royal Infirmary, Glasgow, G31 2ER, United Kingdom

**Keywords:** Colorectal, Dexamethasone, Inflammation, Steroid, Surgery, Perioperative

## Abstract

**Introduction:**

Resectional surgery remains the mainstay of treatment for colorectal cancer. A heightened postoperative systemic inflammatory response has been shown to correlate negatively with short/long-term outcomes. Perioperative steroid administration may help to alleviate this systemic inflammatory response. This survey has been carried out to assess current attitudes towards perioperative steroid use and to gauge interest in a randomised control trial in this area.

**Method:**

An internet-based survey consisting of 9 questions was circulated via email. Those responses from outside the United Kingdom were excluded.

**Result:**

74 doctors from the United Kingdom, predominantly Consultant Anaesthetists (54%) responded to this survey. 77% gave some or all of their patients steroids, in 75% of cases at the discretion of the anaesthetist. The main perceived benefit was to reduce postoperative nausea and vomiting. Diabetics and those deemed at high risk of wound infection were the group in whom most respondents would be reluctant to give steroids. 32% of respondents had no concerns. 87% of respondents felt that a randomised trial in this field would be of clinical interest with most respondents (58%) preferring a three-armed trial – no steroids vs low dose steroids vs high dose steroids.

**Conclusion:**

This survey indicated that perioperative steroid use is currently widespread. Sufficient equipoise exists for a trial in this area with regard to examining the impact of dexamethasone on postoperative complications and the postoperative systemic inflammatory response. Respondents favoured a 3-armed trial – no steroids vs low-dose steroids vs high-dose steroids.

## Highlights

• Currently, the use of perioperative steroids is common, although not routine in resectional colorectal surgery.
• Perioperative steroids are currently administered with the aim of reducing postoperative nausea and vomiting.
• There is sufficient equipoise to carry out an RCT studying perioperative dexamethasone and outcomes in colorectal resection.

## 1 Introduction

Colorectal cancer is the third most commonly diagnosed malignancy worldwide with approximately 1.8 million new cases being diagnosed each year [1]. Within the United Kingdom colorectal cancer accounts for around 16,000 deaths annually [2]. Curative resectional surgery remains the mainstay of treatment for this condition though carries with it not only a significant risk of overall morbidity and mortality but also a significant risk of cancer related mortality even after curative treatment.

The relationship between an increased postoperative systemic inflammatory response and increased likelihood of complications has now been well documented within the medical literature [[3], [4], [5]] as has the relationship between the postoperative systemic inflammatory response, postoperative complications and long-term oncological outcome and survival [[5], [6], [7]]. The potential to modulate the postoperative systemic inflammatory response is therefore of clinical interest as one would hypothesise that this may reduce postoperative morbidity and mortality and improve long-term cancer free survival.

Single dose corticosteroids are frequently administered in the perioperative period to reduce postoperative nausea and vomiting [[8], [9], [10]]. A previous retrospective propensity matched observational study from our department that included patients undergoing elective resectional surgery for colorectal cancer demonstrated a reduction in both the magnitude of the postoperative systemic inflammatory response and the overall complication rate in those patients who received single dose dexamethasone on induction of anaesthesia [11]. While a recent large randomised control trial [10] documented the beneficial effect of single dose dexamethasone on postoperative nausea and vomiting it did not measure the postoperative systemic inflammatory response. The administration of preoperative corticosteroids appears to be safe with a previous meta-analysis [12] not demonstrating any association between preoperative corticosteroid administration and an increased risk of any complications including anastomotic leaks (p = 0.79).

This qualitative questionnaire-based study aimed to examine both attitudes towards and current practice of perioperative steroid administration in resectional colorectal surgery. This would improve knowledge of current perioperative steroid use and perceived benefits and risks of this adjunct to anaesthesia. It would also help to assess whether there is current equipoise to carry out further research in this area including the effect of perioperative steroid use on the postoperative systemic inflammatory response, morbidity and mortality within the context of curative colorectal cancer surgery.

## 2 Methods

This qualitative analysis utilised an internet based survey which was generated on the SurveyMonkey website [13] and included 9 questions regarding the use of perioperative steroids in resectional colorectal cancer surgery (Table 1).

**Table 1:** Questions included in online survey.

**Question 1: In which country/region do you work in?**	
a. United Kingdom	d. Australia
b. Europe	e. Other (please specify)
c. USA	
**Question 2: What is your grade and speciality?**	
a. Anaesthetist (Consultant)	e. Consultant Surgeon (Non-Colorectal)
b. Anaesthetist (Trainee)	f. Career Grade Surgeon
c. Anaesthetist (Other)	g. Surgical Trainee
d. Consultant Surgeon (Colorectal)	h. Other (please specify)
**Question 3: Do any of your patients receive steroids in the perioperative period? If yes, please specify the drug and dose or unknown if unsure.**	
a. Yes (all)	c. No
b. Yes (some)	a. Unsure
**Question 4: If you answered yes to Question 3 who makes the decision regarding administration of steroids? If you answered no to Question 3 please select N/A**	
a.Surgeon	d.Part of ERAS or similar protocol
b.Anaesthetist	e.N/A
c.Combination	
**Question 5: If you answered yes to Question 3 when are steroids given to patients? If you answered no to Question 3 please select N/A**	
a.Preoperatively	c.Post-operatively
b.Intra-operatively/on induction of anaesthesia	d.N/A
**Question 6: Are there any groups of patients to whom you would be reluctant to give perioperative steroids? (Select all that apply)**	
a.Diabetics (diet/tablet controlled)	d.High risk of wound infection
b.Diabetics (insulin controlled)	e.Emergency cases
c.Renal failure	f.Other (please specify)
**Question 7: Do you think that single dose steroids given perioperatively for colorectal cancer are associated with? (Select all that apply)**	
a.Reduced postoperative nausea/vomiting	e.Reduced postoperative mortality
b.Reduced overall complications	f.Improved long term survival
c.Reduced anastomotic leaks	g.None of the above
d.Reduced surgical stress response	h.Other (please specify)
**Question 8: Do you have concerns regarding the use of perioperative single dose steroids for patients undergoing colorectal cancer resection? (Select all that apply)**	
a.Diabetes/hyperglycaemia	f.Adrenal complications
b.Increased wound complications	g.Gastric complications
c.Increased anastomotic leak rate	d.Renal failure
e.Psychiatric/mood problems	f.No concerns
g.Insomnia	h.Other (please specify)
**Question 9: If a trial was set up examining steroid administration at induction of anaesthesia for colorectal resection, with the aim of addressing the effect on the postoperative systemic inflammatory response and complications, do you think there would be sufficient equipoise to recruit for the following: (Please select all that you feel would be of interest)**	
a.2-Armed trial – steroids vs no steroids	c.3-Armed trial – no steroids vs low dose steroids vs high dose steroids
b.2-Armed trial – low vs high dose steroids	d.Insufficient equipoise for a trial in this area

Once this survey was generated, the link to it was publicised on our department's Twitter feed. Additionally, a list of email addresses was obtained for surgeons and anaesthetists who have published in this area and the access link was circulated to them. As only a minority of participants were from outside the United Kingdom we decided to exclude these to provide insight into current use of and attitudes towards perioperative steroid administration in the United Kingdom.

The survey was circulated in February 2019 and closed for responses in March 2019. No incentives were used to encourage participation. Data was analysed and graphs were created using Microsoft Office and IBM SPSS Statistics Version 24. This study has been registered with the Research Registry – unique identifier “researchregistry5124”. There are no conflicts of interest or sources of funding to declare. All responses to this survey were anonymous.

Numerical data has been displayed as numbers and/or percentages to the nearest whole number. The variation in responses between surgeons and anaesthetists have been compared using the Chi squared test with a two tailed p value of <0.05 being considered statistically significant.

## 3 Results

Overall, 100 people responded to this survey. 97 people answered Question 1, 76% (n = 74) of whom worked in the UK - only these responses were included in further analysis.

74 people answered Question 2. 54% of respondents were Consultant Anaesthetists, 22% Consultant Colorectal Surgeons, 11% Anaesthetic Trainees, 9% Surgical Trainees and 4% Consultant Non-Colorectal Surgeons.

70 people answered Question 3. 54% of respondents give some patients steroids in the perioperative period, 23% give all patients steroids in the perioperative period, 14% do not give any patients steroids in the perioperative period and 9% of respondents were unsure whether their patients receive steroids or not. 48 people provided a free text comment regarding choice of steroids with greater than 90% using dexamethasone. The majority of these who specified a dose administer between 3.3 mg and 8 mg of dexamethasone perioperatively.

70 people answered Question 4. Of those whose patients receive steroids, the decision regarding perioperative steroid use was made by anaesthetists in 75% of cases, is protocol driven in 12% of cases, made by a combination of surgeons and anaesthetists in 11% of cases and made by surgeons in 2% of cases. The remainder do not use perioperative steroids.

70 people answered Question 5. Of those who give perioperative steroids, 90% of respondents administer steroids intraoperatively/on induction of anaesthesia, 5% give steroids preoperatively and 5% give steroids postoperatively.

**Fig. 1:**
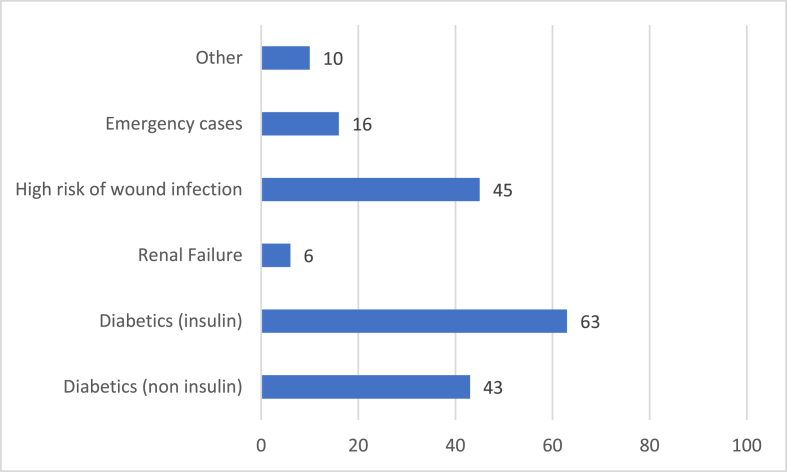
Are there any groups of patients to whom you would be reluctant to give perioperative steroids?.

67 people answered Question 6 (Fig. 1). 63% of respondents would be reluctant to give steroids to insulin-controlled diabetics, 45% to those deemed at high risk of wound infection, 43% to other diabetics, 16% to emergency cases and 6% to patients with renal failure. 10% would be reluctant to give steroids to other patients including those who were septic or at high risk of delirium.

**Fig. 2:**
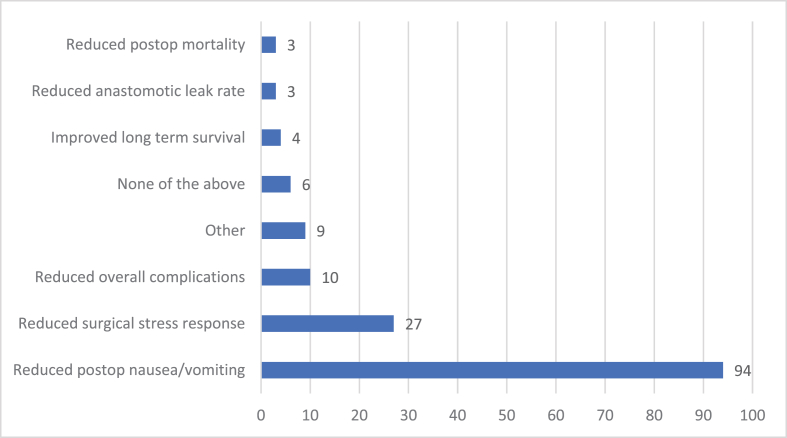
Do you think that single dose steroids given perioperatively for colorectal cancer are associated with.

67 people answered Question 7 (Fig. 2). 94% of respondents think that perioperative steroids reduce postoperative nausea and vomiting, 27% think they reduce the surgical stress response, 10% think they reduce overall complications, 6% think they have no beneficial effect, 4% think they improve long term survival, 3% think they reduce anastomotic leak rate and 3% think they reduce postoperative mortality. 9% of respondents think that they had other beneficial effects which, based on free text responses, were predominantly analgesic effects.

**Fig. 3:**
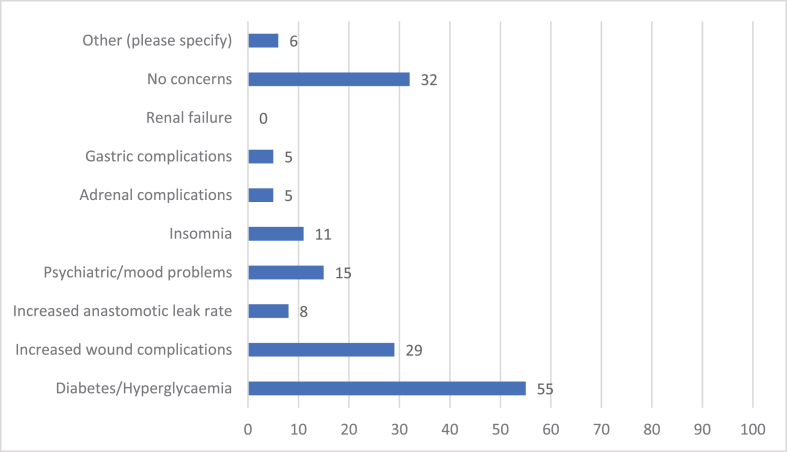
Do you have any concerns regarding the use of perioperative single dose steroids for patients undergoing colorectal cancer resection?.

66 people answered Question 8 (Fig. 3). 55% of respondents would be concerned about uncontrolled diabetes/hyperglycaemia, 29% about increased wound complications, 15% about increased psychiatric/mood disturbance, 11% about insomnia, 8% about increased anastomotic leak rate, 5% about increased adrenal complications and 5% about increased gastric complications. 6% of responders had other concerns – based on free text comments this was predominantly concern over long term oncological outcomes.

**Fig. 4:**
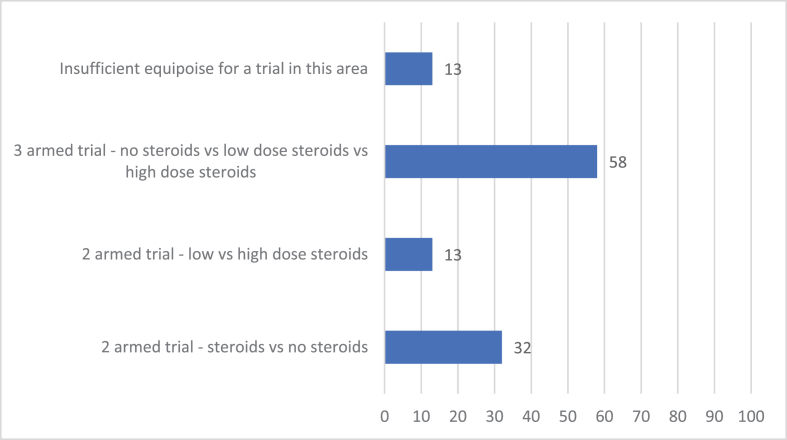
If a trial was set up examining steroids administered at induction of anaesthesia, do you think there would be sufficient equipoise to assess the following?.

60 people answered Question 9 (Fig. 4). 87% of responders think there would be sufficient equipoise for a trial in this area. 58% think there would be sufficient equipoise for a 3-armed trial (no steroids vs low dose steroids vs high dose steroids), 32% for comparison of steroids vs no steroids and 13% for low vs high dose steroids. 13% of responders do not think there would be sufficient equipoise for a trial in this area.

As shown in Table 2 when the responses of surgeons (any grade) were compared to anaesthetists (any grade) significantly more anaesthetists were concerned about giving steroids to non-insulin diabetics (58% vs 14%, p = 0.001) and insulin dependent diabetics (80% vs 27%, p < 0.001). Significantly more anaesthetists were concerned about uncontrolled diabetes/hyperglycaemia following steroid administration (73% vs 14%, p < 0.001) and a higher proportion of surgeons were concerned about the effect of steroids on anastomotic leak rate (18% vs 2%, p = 0.021). Significantly more surgeons than anaesthetists had either no concerns or other concerns regarding steroid administration (55% vs 18% p = 0.002).

**Table 2:** Responses to views of perioperative steroids in surgeons (any grade) compared to anaesthetists (any grade).

	Surgeon	Anaesthetist	P
**Groups of patients reluctant to give steroids**			
Non-insulin dependent diabetes	3 (14%)	26 (58%)	0.001
Insulin dependent diabetes	6 (27%)	36 (80%)	<0.001
Renal failure	2 (9%)	2 (4%)	0.451
High risk wound infection	7 (32%)	21 (47%)	0.247
Emergency cases	1 (5%)	0 (0%)	0.150
Other	2 (9%)	3 (7%)	0.723

**Associations of steroids**			
Reduced postop nausea/vomiting	19 (86)	44 (98%)	0.064
Reduced overall complications	3 (14%)	4 (9%)	0.551
Reduced anastomotic leaks	1 (5%)	1 (2%)	0.600
Reduced surgical stress response	8 (36%)	10 (22%)	0.220
Reduced postop mortality	1 (5%)	1 (2%)	0.600
Improved long term survival	2 (9%)	1 (2%)	0.202
None of the above	1 (5%)	0 (0%)	0.150
Other	0 (0%)	3 (7%)	0.215

**Concerns regarding steroids**			
Diabetes/hyperglycaemia	3 (14%)	32 (73%)	<0.001
Increased wound complications	4 (18%)	15 (34%)	0.178
Increased anastomotic leak rate	4 (18%)	1 (2%)	0.021
Psychiatric/mood problems	1 (5%)	9 (21%)	0.089
Insomnia	3 (14%)	4 (9%)	0.572
Adrenal complications	1 (5%)	2 (5%)	1
Gastric complications	2 (9%)	1 (2%)	0.210
Renal failure	0	0	X
Other	12 (55%)	8 (18%)	0.002
No concerns	12 (55%)	8 (18%)	0.002

**Equipoise for trial**			
Steroids vs no steroids	5 (25%)	14 (34%)	0.469
Low vs high dose steroids	5 (25%)	2 (4.9%)	0.021
No steroids vs low dose vs high dose	14 (70%)	22 (53.7%)	0.223
No equipoise for trial	3 (15%)	4 (9.8%)	0.546

## 4 Discussion

The present study was predominantly completed by Consultant Anaesthetists working in the United Kingdom. The majority of respondents gave some/all of their patients intraoperative steroids with the decision of whether to administer steroids being at the discretion of the anaesthetist in most cases. Reduction of postoperative nausea and vomiting was the primary aim of perioperative steroid administration. Reluctance to administer steroids was particularly notable for those patients who are diabetic, particularly insulin dependent and those at high risk of wound infection. This study suggests that there is sufficient equipoise to carry out a randomised control trial examining the impact of dexamethasone on the postoperative systemic inflammatory response and complications following colorectal resection with respondents indicating that a three-armed trial comprising no steroids vs low dose steroids vs high dose steroids would be the preferred format for this.

Based on this survey there is concern regarding the use of perioperative single dose dexamethasone within the diabetic cohort of patients, particularly those with insulin dependent diabetes. Two recent randomised control trials [14,15] have reported a significant increase in blood glucose levels following dexamethasone administration in both diabetic and non-diabetic patients although there was no significant difference in the size of the effect between diabetics/non-diabetics in either study. A recent Cochrane review [16]. Similarly reported an increased blood glucose following dexamethasone however evidence was limited and there was no evidence of increased negative outcomes as a result of this although diabetic patients were excluded from most trials.

A previous meta-analysis [12] analysed the impact of corticosteroids on both postoperative complications and the postoperative systemic inflammatory response. It reported a significant reduction in both overall complications and postoperative systemic inflammatory response in those patients receiving preoperative corticosteroids although a significant reduction in infective complications was seen following surgery for liver but not colorectal malignancy. Similarly, a recent propensity matched cohort study [11] which included patients undergoing surgery for colorectal cancer reported a significantly lower postoperative systemic inflammatory response and overall complications in those patients receiving preoperative dexamethasone. A further randomised trial [17] of 73 patients undergoing maxillofacial surgery reported a significant reduction in postoperative CRP rise within the cohort receiving preoperative dexamethasone.

While a recent large multicentre randomised trial (DREAMS) [10] reported a significant reduction in postoperative nausea and vomiting in patients undergoing elective bowel surgery who received single dose perioperative dexamethasone it did not investigate the difference in postoperative systemic inflammatory response between groups. Although not limited to colorectal surgery, the PACMAN trial [18] is currently underway and will include surgical complications. However, while CRP levels will be collected, postoperative levels of inflammation are not included as either primary or secondary outcomes. The PADDI trial [19] is also currently underway and will include surgical site infection as a primary outcome, however this trial neither includes postoperative inflammation as a primary outcome, nor is limited to colorectal resections.

Limited literature exists reporting the effect of steroid use on long-term disease-free survival. One study comparing 515 patients with stage I-III rectal cancer did report, on multivariate analysis, a reduction in disease-free and overall survival in those patients receiving intravenous dexamethasone A follow up study [20] of a previously conducted small trial of 43 patients undergoing colonic resection also reported a higher rate of distant recurrence in those patients receiving dexamethasone. However, a recently conducted large propensity matched study [21] of 2729 patients undergoing breast cancer surgery did not find an association between perioperative dexamethasone administration and increased recurrence or mortality. Furthermore, an observational study [22] of 679 patients undergoing pancreaticoduodenectomy for cancer reported improved overall survival in patients given intraoperative dexamethasone.

The present study has a several limitations. This survey is small, particularly for the subgroup of analysis comparing responses between surgeons and anaesthetists. To encourage participation in the survey we intentionally kept survey length short and questions simple. Furthermore, medical professionals who have a greater interest in either perioperative steroid use and evidence based medical practice or have stronger opinions either for or against perioperative steroid use were more likely to respond and therefore may bias results.

It would be of interest to examine within a randomised control trial the effect of steroid administration at induction of anaesthesia on the postoperative systemic inflammatory response and postoperative short/long-term outcomes in patients undergoing a curative resection for colorectal cancer. An observational study by McSorley et al., 2019 [23] suggested a dose dependent effect of dexamethasone with higher doses of dexamethasone being associated with a reduced postoperative systemic inflammatory response and postoperative complications. Therefore, a trial which incorporated a low-dose steroid arm (e.g. dexamethasone 4 mg) and high-dose steroid arm (e.g. dexamethasone 8 mg) would be worthwhile.

In summary, this study has suggested that while most patients currently receive perioperative steroids with the primary aim of reducing postoperative nausea and vomiting (PONV) they are not used routinely for all patients. Furthermore, no consensus exists as to the correct dose, even in the context of the prevention of PONV. Sufficient equipoise appears to exist for a randomised control trial in colorectal cancer resection, with a three-armed RCT comparing no steroids vs low dose steroids vs high dose steroids being the most popular choice of design. Ideally, anaesthetic technique would be otherwise standardised. In our opinion such a study should include postoperative complications as the primary outcome and a measure of the postoperative systemic inflammatory response (e.g. C-reactive protein) as a secondary outcome. If included, diabetic patients and those at increased risk of wound infection would need close monitoring to ensure that they did not have an unacceptably increased risk of complications. Additionally, long term follow up of patients included in such a trial would be important, to identify whether perioperative single dose steroid usage alters disease free survival, although if not powered for this outcome then it may remain an area of uncertainty.

## Ethical approval

Not required.

## Sources of funding

Nil.

## Author contribution

All authors contributed equally to design and writing of study.

## Trial registry number – ISRCTN

N/a.

## Guarantor

Allan Golder.

## Research registration unique identifying number (UIN)

N/a.

## Provenance and peer review

Not commissioned, externally peer reviewed.

## Declaration of competing interest

None to declare.
